# Formation of Anisotropic Conducting Interlayer for High‐Resolution Epidermal Electromyography Using Mixed‐Conducting Particulate Composite

**DOI:** 10.1002/advs.202308014

**Published:** 2024-04-10

**Authors:** Zifang Zhao, Han Yu, Duncan J. Wisniewski, Claudia Cea, Liang Ma, Eric M. Trautmann, Mark M. Churchland, Jennifer N. Gelinas, Dion Khodagholy

**Affiliations:** ^1^ Department of Electrical Engineering Columbia University New York 10027 USA; ^2^ Department of Biomedical Engineering Columbia University New York 10027 USA; ^3^ Department of Neuroscience Columbia University New York NY 10032 USA; ^4^ Zuckerman Mind Brain Behavior Institute Columbia University New York 10027 USA; ^5^ Kavli Institute for Brain Science Columbia University New York 10032 USA; ^6^ Grossman Center for the Statistics of Mind Columbia University New York USA; ^7^ Department of Neurology Columbia University Irving Medical Center New York 10032 USA; ^8^ Department of Electrical Engineering University of California Irvine CA 92697 USA

**Keywords:** anisotropic conductors, conducting polymers, EMG, organic bioelectronics

## Abstract

Epidermal electrophysiology is a non‐invasive method used in research and clinical practices to study the electrical activity of the brain, heart, nerves, and muscles. However, electrode/tissue interlayer materials such as ionically conducting pastes can negatively affect recordings by introducing lateral electrode‐to‐electrode ionic crosstalk and reducing spatial resolution. To overcome this issue, biocompatible, anisotropic‐conducting interlayer composites (ACI) that establish an electrically anisotropic interface with the skin are developed, enabling the application of dense cutaneous sensor arrays. High‐density, conformable electrodes are also microfabricated that adhere to the ACI and follow the curvilinear surface of the skin. The results show that ACI significantly enhances the spatial resolution of epidermal electromyography (EMG) recording compared to conductive paste, permitting the acquisition of single muscle action potentials with distinct spatial profiles. The high‐density EMG in developing mice, non‐human primates, and humans is validated. Overall, high spatial‐resolution epidermal electrophysiology enabled by ACI has the potential to advance clinical diagnostics of motor system disorders and enhance data quality for human‐computer interface applications.

## Graphical abstract

Biocompatible, anisotropic‐conducting interlayer composites (ACI) that establish an electrically anisotropic interface with the skin are developed. These composites significantly enhance the spatial resolution of epidermal electromyography (EMG), enabling the application of dense cutaneous sensor arrays and facilitating the acquisition of single muscle action potentials.

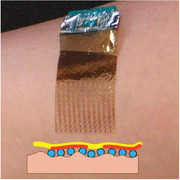

## 1 Introduction

Non‐invasive acquisition of electrophysiological activity from the surface of the skin permits facile clinical diagnostics with minimum procedural risk. These recordings can be employed for the majority of electrically active tissues such as the brain, nerves, heart, and muscles.^[^ ^1^, ^2^, ^3^, ^4^, ^5^ ^]^ In parallel, there is a high demand for expanding the applicability of such epidermal approaches by improving the spatiotemporal resolution of the recordings.^[^ ^6^, ^7^ ^]^ Although it is possible to perform electromyography (EMG) from the surface of the skin, clinical EMG is predominantly obtained using intramuscular needle electrodes for diagnosis of conditions such as denervation, myopathy, or muscular dystrophy.^[^ ^8^, ^9^, ^10^ ^]^ This skin penetrating technique has been necessary to ensure recording from specific, sometimes small, muscles at the resolution of individual muscle action potentials. With the development of integrated multi‐channel electrophysiology circuits, low‐amplitude EMG signals could be recorded across the epidermis overlying muscles, enabling signal localization and correlation with behavior and movements.^[^ ^11^, ^12^ ^]^ Despite the promising potential of epidermal EMG, the spatiotemporal limits of this method remain unclear due to the complex, multi‐node impedance circuity between electronics and sub/cutaneous tissue.^[^ ^13^, ^14^, ^15^ ^]^

Higher temporal and spatial resolution recordings can be achieved by using smaller and denser electrodes due to more confined charge integration on the surface of the electrode.^[^ ^7^ ^]^ However, reducing electrode size results in higher electrochemical impedance at the electrode/tissue interface due to the reduced area of electric double‐layer (EDL) capacitance.^[^ ^16^, ^17^, ^18^ ^]^ Non‐polarizable conducting polymers can address this challenge by lowering the electrochemical impedance of the electrodes through their mixed conduction and large effective surface area.^[^ ^16^, ^19^ ^]^ However, these materials are in a solid state and cannot establish a reliable mechanical interface with the skin independently without the presence of an adhesion layer.^[^ ^20^ ^]^ Therefore, to establish an interface with low electrochemical impedance and reliable mechanical stability, ionic‐conducting gels or pastes are often incorporated as an interlayer between electrodes and skin.^[^ ^21^, ^22^ ^]^ They can significantly improve this interface and often are necessary for clinical EEG studies. However, the use of such interface layers is limited to protocols with sparse electrode placements because they can establish an unwanted conduction path between electrodes resulting in inter‐electrode crosstalk and diminished spatial resolution.^[^ ^23^ ^]^ Conformable and stretchable electronics that can adapt to muscle contraction and follow the curvilinear surface of the skin can significantly improve the electrode/tissue interface.^[^ ^24^, ^25^, ^26^ ^]^ Moreover, their light weight effectively decouples the interface from mechanical forces induced by rigid electronics. Electrodes within such devices are often times made with porous or mixed‐conducting materials to further improve the interface.^[^ ^12^, ^27^, ^28^, ^29^, ^30^, ^31^ ^]^ However, they are dry and are not as robust as gel interlayers in suppressing mechanical noise. In addition, the application of such materials to skin areas with high hair density could lower the quality of the interface, requiring electrode size to be increased.

Here, we show that a combination of conformable electronics and biocompatible, anisotropic‐conducting interlayer composites (ACI) can improve the spatiotemporal resolution of EMG recording to the level of acquiring individual muscle action potentials. We characterized the electrode/tissue interface using 3 and 4 port in vivo electrochemical impedance spectroscopy (EIS) and demonstrated the impact of an ion‐conducting interlayer on the spatial resolution of EMG recordings. We then established an anisotropic interface between electrodes and tissue using ACI. We demonstrated the improved spatial resolution of EMG recording in infant mice, non‐human primates, and human subjects. These recordings yielded putative single motor units (MUs) that had a temporal correlation with muscle activity during behavioral tasks. Lastly, we were able to accurately decode and reconstruct continuous finger movement based on the firing rate of MUs. Our results support the notion that ACI could broaden the applicability of epidermal EMG and has the potential to provide new opportunities for accurate, minimally invasive brain machine interfaces.

## 2 Results

**Figure 1:**
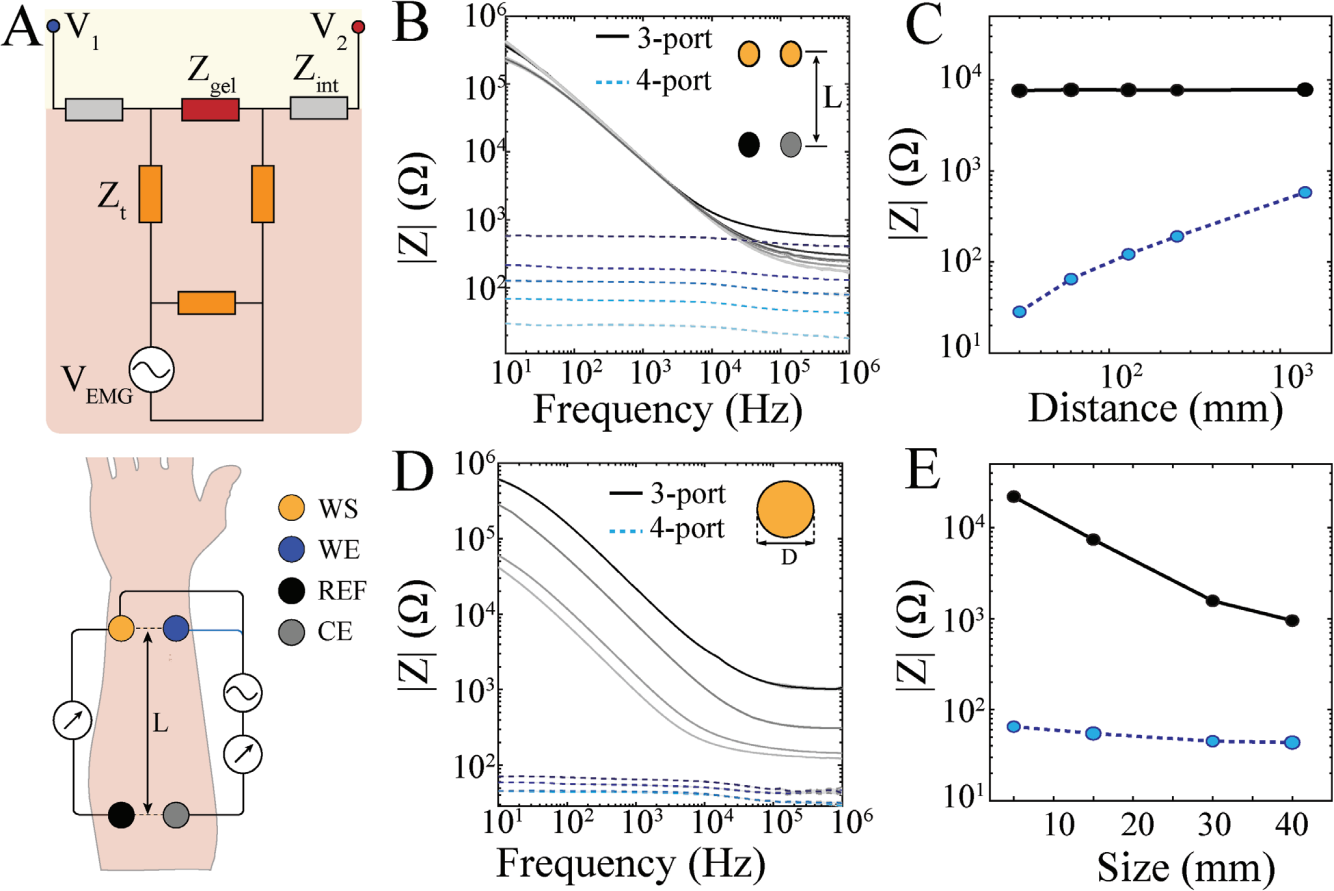
EIS of cutaneous tissue. A) Schematic cross‐sectional circuit model demonstrating an EMG voltage source (V_EMG_) and two overlying recording electrodes. Z_int_, Z_Gel_ and Z_T_ denote electrode/tissue interface, ion‐conducting interlayer, and subcutaneous tissue impedances, respectively (top). Diagram of electrode placement. WE: work electrode, WS: work sense electrode, REF: reference electrode, CE: counter electrode; L marks the distance between WE and REF electrodes (bottom). B) In vivo EIS using 3‐ (black) and 4‐port (blue) configuration with various distances between electrodes (L). Darker shades represent shorter distances. C) Bulk tissue impedance (4‐port; blue) varies with electrodes distance whereas electrode/tissue interface (3‐port; black) impedance remains constant. Impedance values were taken at 1 kHz. D) In vivo EIS using 3‐ (black) and 4‐port (blue) configuration with various electrode sizes (D). Darker shades represent smaller sizes. E) Electrode/tissue interface (3‐port; black) impedance varies with electrode size whereas bulk tissue impedance (4‐port; blue) remains constant. Impedance values were taken at 1 kHz

The signal‐to‐noise ratio (SNR) and spatiotemporal resolution of EMG recordings are functions of electrode number, location, spacing, impedance and their material composition at the interface with biological tissue.^[^ ^32^ ^]^ To investigate these parameters, we constructed a simplified equivalent circuit model of two adjacent EMG electrodes (E_1_ and E_2_) consisting of an EMG voltage source (V_EMG_) originating from muscle fibers, and impedances arising from the electrode/tissue interface (Z_int_), ion‐conducting interlayer (Z_Gel_) and subcutaneous tissue (Z_T_) (**Figure** **1**A, top). Similar to other electrophysiological setups, such as surface or intracranial brain recordings, lowering Z_int_ via improvement of EDL capacitance leads to better transduction of ionic to electronic signals and improved SNR of recordings.^[^ ^33^, ^34^, ^35^ ^]^ Additionally, higher density and number of electrodes could result in improved resolution. However, because Z_Gel_ is shared among densely packed electrodes, we asked what is the contribution of Z_Gel_ to SNR and spatiotemporal resolution of EMG recordings? This interlayer is often composed of highly conducting hydrogels or ionic liquids that enhance SNR by improving the EDL capacitance due to a stable electrode/tissue interface. However, it also serves as a highly conducting medium for the propagation of V_EMG_ to distant electrodes, diminishing spatial resolution. To derive the optimal impedance ranges of these components, we first performed in vivo EIS in 3‐ and 4‐port configurations without the presence of an interlayer to extract Z_int_ and Z_T_. We placed 4 identical disc electrodes (10 mm diameter) on the forearm with varying distance between working (work and sense) and reference electrodes (Figure 1A; bottom). The 3‐port EIS measurements did not vary with distance (3 – 25 cm) in the physiological bandwidth (0.1 Hz–5 kHz), whereas 4‐port measurements were proportional to distance (Figure 1B,C; Figure S1, Supporting Information). Given that 4‐port (i) eliminates the interface impedances, and (ii) is a function of electrode distance, we posited that these measurements reflected the bulk tissue properties.^[^ ^36^, ^37^ ^]^ To further validate this notion, we conducted additional sets of experiments that varied electrode size while maintaining a constant distance between them (Figure 1D,E; Figure S1A,B, Supporting Information). In contrast to the previous experiment, the 3‐port measurements varied by electrode size while the 4‐port impedances remained unchanged. These results suggest that 3‐port impedances contain the interface impedance (Z_int_) and confirm that 4‐port impedances reflect the bulk tissue impedance (Z_T_)_._ We then defined spatial resolution as the ratio between E1 and E2 voltages with V_EMG_ in the vicinity of E1 and connected to E2 via Z_Gel._ This ratio is also indicative of inter‐electrode crosstalk. Highly conducting gels with similar electrical properties to Z_T_ (≈ 100 Ω) introduced a significant potential at V_2_, resulting in crosstalk. We found that to reach a V_1_/V_2_ ratio of at least 10^2^, Z_Gel_ should be larger than 10 kΩ across the physiological frequency range (Figure S1C, Supporting Information). Overall, these results suggest that the ideal interlayer material should be able to i) enhance the electrochemical impedance of Z_int_, ii) provide a highly resistive lateral pathway, and iii) continue to maintain a mechanically stable electrode/tissue interface.

**Figure 2:**
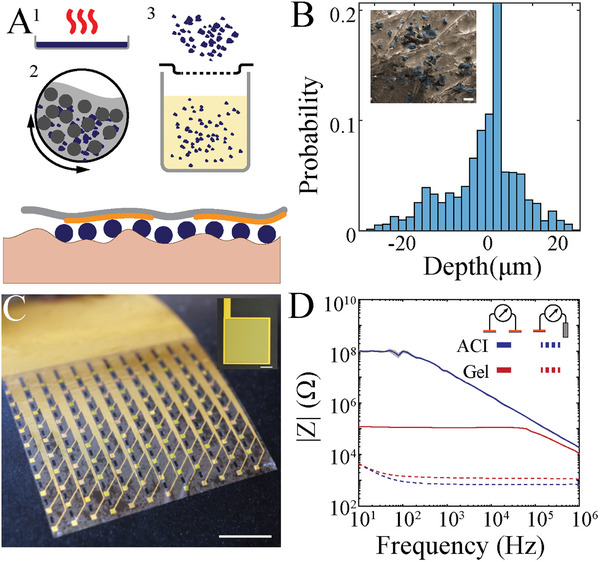
ACI enables robust anisotropic conducting electrode/tissue interface. A) Illustration of ACI fabrication process: 1) conducting flakes are generated by baking a PEDOT:PSS‐based dispersion; 2) conducting flakes are pulverized using an IPA‐based ball mill; 3) particles are filtered and mixed with the adhesive scaffolding polymer (top). Cross‐sectional schematic illustrating ACI particles between skin and electrodes. Note there is no conducting path between neighboring electrodes. B) Histogram of skin roughness measured by 2D profilometry. Inset: tilted SEM of ACI particles applied on the surface of artificial skin. Scale bar, 50 µm. C) Optical micrograph of high‐density NeuroGrid for epidermal electrophysiology. Scale bar, 5 mm. Inset: optical microscopy of NeuroGrid. Scale bar, 100 µm. D) EIS of 450 × 450 µm^2^ PEDOT:PSS electrodes with ACI (dashed blue) and conductive gel (dashed red) interlayers. Inter‐electrode impedance (L = 2 mm) with ACI (solid blue) and conductive gel (solid red) interlayers. Shaded areas represent standard errors (n = 3).

To address these challenges, we created (ACI using a mixed‐conducting particulate composite (MCP).^[^ ^38^, ^39^ ^]^ We embedded poly(3,4‐ethylenedioxythiophene)‐poly(styrenesulfonate) (PEDOT:PSS) particles in an ion‐conducting scaffold comprised of chitosan and D‐sorbitol (**Figure** **2**A). We chose 50 µm particle size to accommodate the surface roughness of skin (Figure S2, Supporting Information) as the thickness of the ACI film is primarily defined by the diameter of the conducting polymer particles. Additionally, the presence of D‐sorbitol in the scaffold acts as a biocompatible adhesive that significantly improves the mechanical stability and adhesion of the interface (Figure S3, Supporting Information). To validate these design elements, we deposited the ACI on phantom human skin and examined the distribution of PEDOT:PSS particles using scanning electron microscopy (SEM) (Figure 2B; inset). We found that PEDOT:PSS particles did not aggregate and were distributed over the entire area. Next, to establish a reliable contact between particles and electrodes, we fabricated a parylene‐C (Pa‐C) ‐based conformable (3 µm‐thick) 10 × 12 array of PEDOT:PSS‐electrodes (NeuroGrid). We performed EIS on individual electrodes with commercially available gel or ACI as the interlayer and Ag/AgCl as the reference (REF) electrode. The impedance values were similar across both interlayers and consistently low, characteristic of the conducting polymer‐based electrodes we employed (Figure 2D; black). To measure the interelectrode impedance, which represents crosstalk, we replaced the Ag/AgCl REF electrode with an adjacent conformable electrode. ACI demonstrated a significantly higher inter‐electrode impedance compared to the gel, corresponding to minimal crosstalk and consistent with values generated by our model (Figure 2D; red).

**Figure 3:**
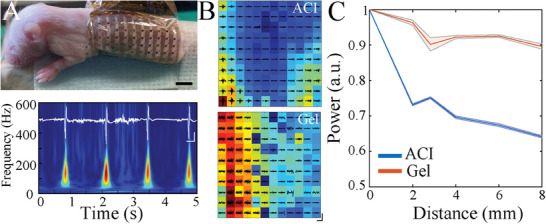
ACI enables high SNR, low‐crosstalk EMG recording in developing rodents. A) Optical image of the mouse pup EMG recording setup. Scale bar, 5 mm (top). Sample time‐frequency spectrogram of epidermal electrical signal showing both ECG and EMG. White trace shows raw acquired data. Scale bar, 50 ms, 200 µV (bottom). B) Comparison of normalized power of band‐passed EMG signal (50–1000 Hz; black) between ACI and conducting gel interlayers. Warmer colors represent higher power. ACI provides a more defined spatial extent (N_ACI _= 1527, N_gel _= 1483 EMG epochs). C) Power coherence of EMG signal as a function of electrode distance. ACI (blue) demonstrates rapid decay in power with distance compared with conductive paste (black). Shaded areas represent standard error (N_ACP_ = 1527, N_gel_ = 1483 EMG epochs).

Muscle activity is an important indicator of motor maturation and sleep stage in developing organisms,^[^ ^40^, ^41^ ^]^ but their small size and fragility typically preclude conventional EMG recording. We tested the ability of devices comprised of PEDOT:PSS electrodes with an ACI interlayer (NeuroGrid‐ACI) to acquire high spatial resolution EMG signals from a commonly used developmental model, the mouse pup. The skin of mouse pups at postnatal day 8 was prepared with either ACI (n = 4) or conductive paste (n = 4) and a NeuroGrid array was placed circumferentially around the torso, covering most of the body surface (**Figure** **3**A, top). Given this NeuroGrid array placement, we monitored both whole‐body EMG and electrocardiography (ECG) signals in an unanesthetized experimental setup (Figure 3A, bottom). ACI preserved spatial information by allowing the identification of more localized, higher amplitude EMG sources compared to the conductive gel (Figure 3B). ACI‐acquired signals also demonstrated a steeper rate of decay with electrode distance (Figure 3C). Therefore, the combination of ACI and NeuroGrid provides enhanced signal spatial resolution in developing organisms.

**Figure 4:**
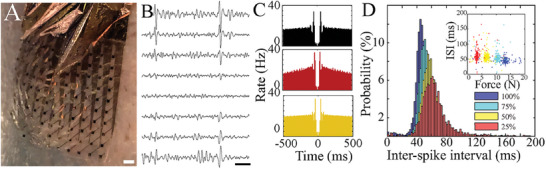
ACI permits non‐invasive detection and clustering of single MU activity from behaving non‐human primates. A) Optical image of electrode array conforming to surface of non‐human primate arm. Scale bar, 2 mm. B) Sample raw traces of non‐human primate EMG. Scale bar, 10 ms. C) Example auto‐correlograms of clustered putative MUs acquired from non‐human primate during an isometric motor task. D) Inter‐spike interval (ISI) distribution of a MU active during four different applied force levels. N_25% _= 1712, N_50% _= 2587, N_75% _= 1987, N_100% _= 1929 spikes. Inset: scatter plot of ISI versus applied force.

EMG is commonly used in adult organisms to monitor movement and muscle activity. We thus applied our ACI‐NeuroGrid to non‐human primates trained to perform a motor task that required the application of variable force to a handheld lever (**Figure** **4**A). Force applied was constantly monitored by a load cell,^[^ ^42^ ^]^ and we acquired localized motor unit (MU) discharges during arm movement. MU activity was identifiable across NeuroGrid electrodes, with a spatial profile that permitted the separation of activity patterns attributable to individual MUs (Figure 4B). Putative single MUs demonstrated distinct refractory periods, confirming appropriate separation and clustering from undifferentiated EMG activity (Figure 4C). As expected, additional MUs were recruited as the amount of muscular force applied increased (Figure S4, Supporting Information). MUs that exhibited early recruitment to the movement also decreased their inter‐spike intervals with increasing force application (Figure 4D). These results indicate that ACI‐NeuroGrid is capable of acquiring EMG on the scale of individual MUs from the surface of the skin during ongoing muscular activity.

**Figure 5:**
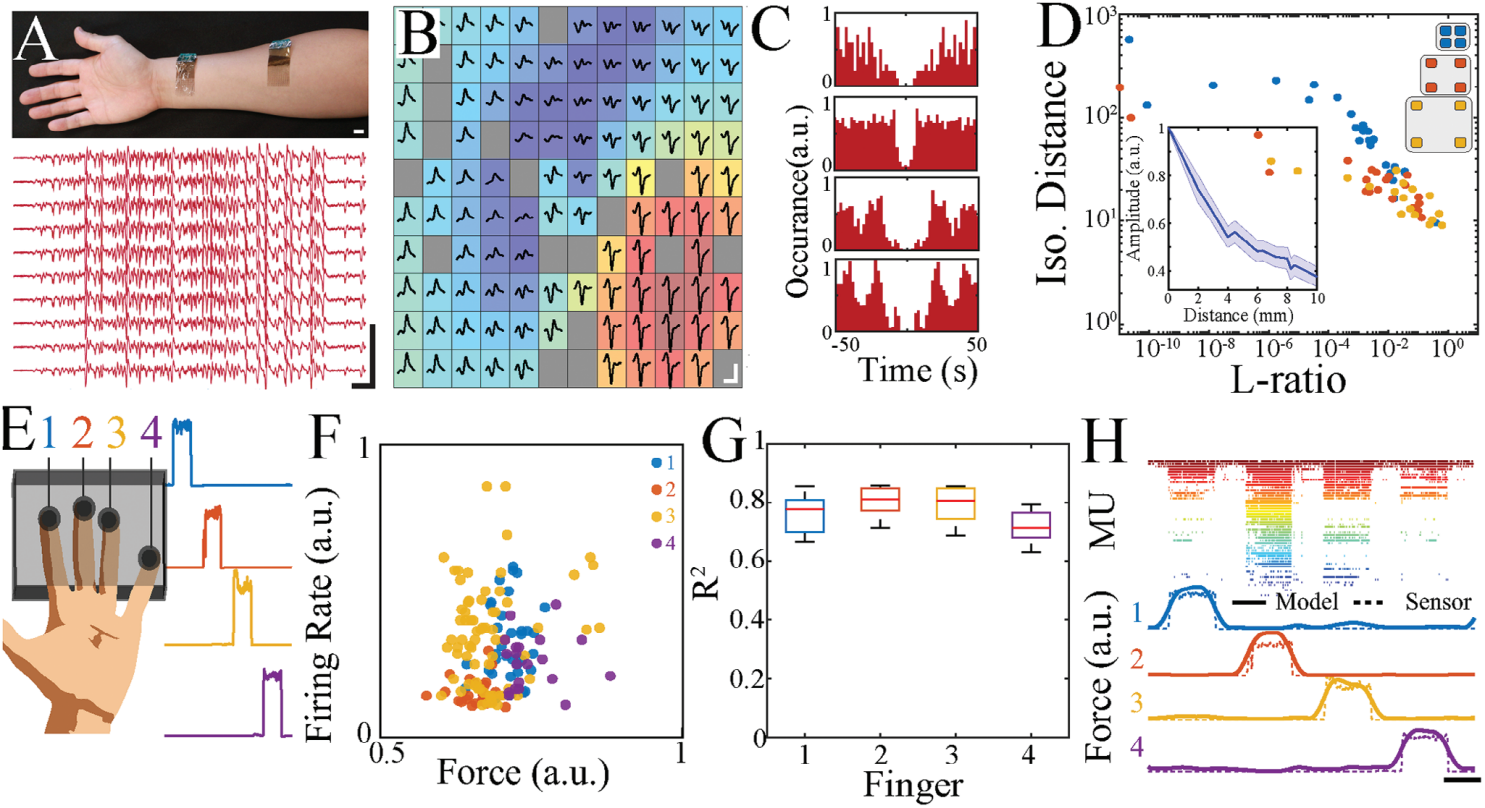
ACI enables capturing of MUs from the surface of the human forearm with accuracy to decode individual finger movements. A) Optical image of NeuroGrids conforming to the forearm of the human subject. Scale bar, 1 cm (top). Sample raw traces of human EMG recorded from the forearm (red). Scale bar, 5 mV, 100 ms (bottom). B) Trigger‐averaged bandpass‐filtered (50‐1000 Hz) EMG traces reveal the spatial distribution of a MU with corresponding power indicated by the superimposed colormap. Scale bar, 100 ms, 1 mV. Channels with insufficient quality for analysis are noted in gray. C) Sample auto‐correlogram (red) of MUs acquired from the surface of forearm. D) Scatter plot of isolation distance versus L‐ratio of MUs with inter‐electrode spacing of 2 mm (blue, n = 46 MUs), 4 mm (red, n = 49 MUs), and 6 mm (yellow, n = 60 MUs). Inset: normalized amplitude of human MUs (n = 22 MUs) as a function of electrode distance. Shaded regions indicate standard error. E) Schematic of the experimental setup showing participant hand inside a 3D‐printed box with four load cells (left). Sample finger pressure measured by load‐cell. Scale bar, 10 s, 20 N (right). Task involved pressing on the load cells in sequence (1: index finger, 2: middle finger, 3: ring finger, 4: pinky). F) Scatter plot of firing rate as a function of force during index (blue), middle (orange), ring (yellow), and pinky (purple) finger pressing epochs. G) Goodness of fit (R^2^) values for measured versus decoded force values across sessions. Binning window = 250 ms. 1: 0.77671 ± 0.021582, 2: 0.81133 ± 0.016429, 3: 0.80792 ± 0.019255, 4: 0.71288 ± 0.017669, N_recording_ = 10 sessions, N_epochs_ = 49986 (Time bins). H) Raster plot of MU activity over the course of 4 sequential finger movements sorted according to the firing rate of the MUs (top). Decoded finger pressure (solid lines) from MU activity compared to actual pressure sensor output (dashed lines; bottom). Scale bar, 10 s.

Next, we recorded from human subjects (n = 5) by placing NeuroGrid‐ACI devices on the inner forearm area and wrist areas to acquire signal from underlying muscles involved in finger flexion (**Figure** **5**A). Spatially localized EMG was recorded, leading to extraction of single MUs from each area (Figure 5B). A subset of MUs had distinct temporal correlations, suggesting a fine motor activation sequence (Figure 5C). Compiling our MUs across species, we determined the overall spatial decrement of MU amplitude with distance (Figure 5D), and confirmed the ability of ACI to enable EMG surface recording that captures behaviorally relevant activity patterns on the millimeter scale. Lastly, to demonstrate enhanced decoding capacity of surface EMG by ACI, we aimed to decode finger movements using the firing rate of MUs recorded from the forearm. Subjects performed a simple finger press test by sequentially pressing each finger of the hand on a load cell that measured real‐time applied force (Figure 5E). Simultaneously, we recorded EMG with NeuroGrid‐ACI devices applied to the inner forearm of the participants. Activity of putative individual MUs was extracted via clustering. The firing rate of individual MUs was strongly correlated with the onset of finger pressing, and distinct MU firing rates were elicited by pressing of different fingers (Figure 5) We input the MU firing rates of each participant into a multivariate regression with a random forest model and variable data binning (100, 250, and 500 ms). 70% of the data were used to train the model, and 30% were used to validate the training result. The decoded signal demonstrated an accurate temporal correlation with movement from four fingers simultaneously (Figure 5G). We validated the detection accuracy by fitting a linear model between the decoded value and true force level for each finger, then compared the goodness of fit for each linear model (Figure S5, Supporting Information). Thus, ACI was able to substantially enhance the resolution of the epidermal EMG and enable acquisition of putative MUs from the skin surface that contain sufficient information to reconstruct continuous muscle movements.

## 3 Discussion

The ability to accurately decode signals from the surface of the skin is a key element of wearable electronics.^[^ ^43^ ^]^ In this study, we developed ACI using conducting polymer particles embedded in ion‐conducting, biocompatible adhesives to enable a high‐density interface between conformable electronics and skin. Modeling and experimental quantification of the skin‐EMG interface revealed the benefits of ACI compared to conventionally utilized homogeneously conducting gel/paste on spatial recording resolution. ACI reduced the crosstalk between adjacent electrodes, enabling high‐spatial resolution recording of surface signals at the level of individual MUs. The fabrication process of ACI is scalable and can be integrated with variety of materials used in high‐density bioelectronics, including PDMS^[^ ^44^ ^]^ and thermoplastic elastomer,^[^ ^45^ ^]^ establishing applicability to a variety of device designs.

The ability to acquire surface EMG is affected by electrode size and pitch. Smaller electrodes can improve EMG signal amplitude^[^ ^46^ ^]^ and reduce crosstalk,^[^ ^47^ ^]^ but at the expense of increased interface impedance. This increased impedance can be ameliorated by using conjugated polymer coating.^[^ ^16^, ^48^ ^]^ Thus, we created an array comprised of PEDOT:PSS electrodes (NeuroGrid), utilizing 2 mm interelectrode spacing. By combining these arrays with ACI, we were able to realize millimeter‐scale recording density in the absence of crosstalk. We used these NeuroGrid‐ACI devices to demonstrate a breadth of applications. These NeuroGrid‐ACI devices enabled large‐scale monitoring of ECG and EMG from fragile developing organisms with high spatiotemporal resolution. Furthermore, we leveraged NeuroGrid‐ACI devices to identify activity patterns of individual MUs from the skin surface of non‐human primates and human subjects. This data provided insight into ongoing motor tasks and was sufficient to decode fine motor activity patterns. In the current work, we adapted an approach used to cluster neural action potentials to classify individual MUs. We then trained a multivariate regression with a random forest model to correlate MU activity and finger movement. To further advance this work, it will be critical to be able to identify putative MUs with real‐time clustering approaches as well as develop models that can classify simultaneous movements of multiple fingers. Our results indicate that this novel material‐based approach has the potential to create sensitive and effective muscle‐computer interfaces.

## 4 Experimental Section

### Materials

PEDOT:PSS (Clevios PH1000) was acquired from Heraeus. Ethylene glycol, chitosan (50–190 kD, 75%–85% deacetylated), (3‐Glycidyloxypropyl)trimethoxysilane (GOPS), 4‐dodecyl benzene sulfonic acid (DBSA), D‐sorbitol (BioUltra ≥99.5%), glycerol, and PBS were purchased from Sigma–Aldrich. Conductive paste (Ten20) was purchased from Weaver and Company. Micro‐90 was purchased from International Products Corporation.

### Device Fabrication

Silicon wafers (100 mm outer diameter, thickness of 500 µm, SSP) were prepared with an anti‐adhesion soap‐based agent (0.3 wt.% Micro‐90 diluted in deionized water) spin‐coated at 1500 rpm to facilitate device lift‐off from the carrier wafer. A 1.8 µm‐thick parylene‐C (Pa‐C) was deposited using SCS Labcoater 2. Metal traces were patterned through a metal lift‐off process by chemically amplified negative photoresist (AZ nLOF 2020). Suss MA6 Mask Aligner was used for exposure and devices were developed in a metal ion‐free (MIF) developer (AZ 300 MIF) for 2 min. A Ti/Au (10/150 nm) layer was deposited by an e‐beam evaporator (Angstrom EvoVac) and lifted by a power‐controlled sonic bath of photoresist remover. The resulting patterned metal layer was insulated by 1.8 µm of Pa‐C with enhanced adhesion using 3‐(trimethoxysilyl)propyl methacrylate (A‐174 silane). A Pa‐C peel‐off technique was used to pattern the PEDOT:PSS. A hard Ti mask and O_2_ dry etching (Oxford Plasmalab 80+; 180 W, 80 sccm O_2_) was used to define the electrodes and pads. PEDOT:PSS solution was spin‐coated twice (650 rpm. then baked at 110 C for 10 min). High‐conductivity PEDOT:PSS was prepared by mixing 80% PEDOT: PSS (v/v), 20% ethylene glycol (v/v), 0.6% DBSA (v/v) and 1% (v/v) of GOPS.

### ACI Preparation

PEDOT:PSS particles were synthesized as previously reported.^[^ ^44^ ^]^ PEDOT:PSS solution was cast onto a glass petri dish and dehydrated overnight at 120 C in the oven. The resulting film was cut into small fragments suspended in isopropyl alcohol (IPA) and milled in a ball‐mill machine with random size assortment of stainless steel beads. The particulate suspension was serially filtered using PET‐mesh cell strainers with pores of 50 µm in diameter. The suspension was allowed to precipitate, and excess IPA was removed. A 40% w/v D‐sorbitol solution was prepared with 2.5% w/v chitosan in acetic acid. PEDOT:PSS particulates were mixed with the chitosan‐sorbitol solution with a 10:1 w/v of PEDOT:PSS. ACI films were applied to a cleaned skin surface, and allowed to air dry (5 min) prior to lamination of the NeuroGrid. NeuroGrids remained attached to the skin surface via this ACI film for the duration of recordings (1‐3 h).

### Device Preparation

A 128‐channel custom‐made amplifier assembly with two 64‐channel electrophysiology amplifier chips (RHD2164, Intan Technologies) was used. The conformable probes were directly bonded to the ball grid array (BGA)‐based contacts of the amplifier circuit board using MCP.

### Electrochemical Impedance Spectroscopy

EIS measurements were performed with a potentiostat (Gamry Reference 600+, Gamry Instruments). Electrochemical impedance was measured in potentiostatic mode with 100 mV RMS and frequency range from 1 Hz to 5 MHz (10 points per decade). EIS data was then analyzed in Echem Analyst (Gamry Instruments) with a pre‐defined impedance model (CPE with Diffusion). Subsequent statistics and plotting were performed in Matlab (Mathworks).

### Scanning Electron Microscopy

Human skin replicas were created with silicone rubber (Ecoflex 00–20, Smooth‐On, Inc). After curing, the artificial skin was cut into 10 × 10 mm^2^ pieces and then coated with ACI. The coated skin replica was then baked in 60 C for 1 h in the oven to dehydrate. This step was required to permit electron microscopy. Samples were moved to a metal platform and sputtered with 10 nm platinum (Cressington 108). SEM was then performed (Nova NanoSEM 450, FEI).

### Mechanical Characterization

The guidelines outlined by the ASTM standard for the measurement of peel force in the experiments. This includes adhering to the recommended sample preparation, test setup, and testing conditions specified in the standard. A thin layer of ACI was blade‐coated onto a glass slide. Then, a 0.75″ wide polyimide (kapton) film was laminated on the slide. One end of the film was attached to the Nextech Digital Force Gauge. The force gauge was attached to a motorized three‐axis micro‐manipulator (Thorlabs). The 90° pulling test was performed at a ramp rate of 1 mm min^−1^ for a distance of 30 mm.

### Rodent Recording

All animal experiments were approved by the Institutional Animal Care and Use Committee at Columbia University. Five Swiss‐Webster mouse pups (5–9 g, 8 days of age) were used for EMG recording. Pups were kept on a regular 12–12 h light‐dark cycle and housed with the mother prior to recording. No prior experimentation had been performed on these mice. Anesthesia was induced by isoflurane prior to the electrode placement. ACI or conductive paste was applied on the skin surface, followed by placement of the NeuroGrid. Pups were then comfortably positioned, provided with familiar nesting olfactory cures, and placed in an electrically insulated box with temperature and humidity control. Electrophysiology data were sampled at 20 kHz with a commercial electrophysiology acquisition system (RHD2000, Intan Technologies).

### Non‐Human Primate Recording

All animal experiments were approved by the Institutional Animal Care and Use Committee at Columbia University. EMG recording were performed on a male adult macaque monkey (Macaca mulatta). Detailed behavior protocols have been previously published.^[^ ^42^ ^]^ The non‐human primate subject was trained to play an isometric “Pac‐Man” game by controlling a handle with the right arm, and the force applied to the handle was constantly monitored by a load cell (PN FSH01673, FUTEK). The skin of the subject by removing the hair with a trimmer, then applied ACI and NeuroGrid. The voltage signal of the load cell was monitored simultaneously with the electrophysiology recording.

### Human Recording

All experiments were performed in compliance with the Institutional Review Board at Columbia University. Informed consent was obtained from all participants. EMG recording was performed on five healthy volunteers (four males, one female, all right‐handed). One electrode array was placed on the right forearm, and a second electrode array was placed on the inner wrist area of the subject. Four 50N load‐cells (FX29, TE Connectivity Measurement Specialties) were mounted inside a 3D printed box, which was fixed to the table. Participants placed their hand inside the box and pressed the load‐cell in sequence (index, middle, ring and pinky fingers), with 30 s pressing time and 10 s inter‐press resting time.

### Data Processing

Channels with identifiable artifacts and high root mean square (rms) noise were manually rejected, and the remaining EMG signal channels were preprocessed with a custom Matlab script and resampled to 4000 Hz. The motor unit detection was performed using a median‐based threshold (4 × *abs*(data)/0.6745) with a spike clustering algorithm (Kilosort3), and manual curation was performed afterward. For EMG onset detection, the original EMG signal was band‐pass filtered (50–1000 Hz), then extracted with an amplitude threshold detecting algorithm. For time‐frequency analysis, the signal was whitened and spectral analyses were generated using a Gabor‐based analytical wavelet. Decoding was performed using a random forest multivariable regressor implemented in Python with scikit‐learn. The firing rate of motor units and load‐cell data were first normalized and then binned into 100, 250, and 500 ms windows before fitting the model. The models were trained on 70% of the trials and evaluated on the remaining 30%. A grid‐search algorithm was employed to determine the optimal model parameters. The accuracy of each model was assessed using the goodness of fit value (R^2^).

## Conflict of Interest

The authors declare no conflict of interest.

## Author Contributions

D.K. and J.N.G. conceived the project. D.K., Z.Z., D.J.W., and C.C. designed, developed, fabricated, and characterized materials and devices. Z.Z, D.J.W., and C.C. fabricated EMG probes. Z.Z. performed the electrical and electrochemical measurements. L.M. and Z.Z. performed the rodent experiments. D.K., H.Y., and ET performed non‐human primate experiments. Z.Z. and H.Y. performed human experiments. D.K., J.N.G., and Z.Z. wrote the manuscript with input from all authors.

## Supporting information

Supporting Information

## Data Availability

The data that support the findings of this study are available from the corresponding author upon reasonable request.
