# Supplementary material for: Formation of Anisotropic Conducting Interlayer for High‐Resolution Epidermal Electromyography Using Mixed‐Conducting Particulate Composite
Source: Adv Sci (Weinh). 2024 Apr 10;11(27):2308014. doi: 10.1002/advs.202308014 (PMC11251554; doi:10.1002/advs.202308014)
Supplement: Supplementary file 1 — Supporting Information [file ADVS-11-2308014-s001.pdf]

ADVANCED SCIENCE
Open Access

Supporting Information

for *Adv. Sci.*, DOI 10.1002/advs.202308014

Formation of Anisotropic Conducting Interlayer for High-Resolution Epidermal Electromyography Using Mixed-Conducting Particulate Composite

Zifang Zhao, Han Yu, Duncan J. Wisniewski, Claudia Cea, Liang Ma, Eric M. Trautmann, Mark M. Churchland, Jennifer N. Gelinas and Dion Khodagholy**

Formation of anisotropic conducting interlayer for high-resolution epidermal electromyography using mixed-conducting particulate composite

Zifang Zhao[1], Han Yu[1], Duncan J. Wisniewski[1], Claudia Cea[1], Liang Ma[2], Eric M. Trautmann[3,4], Mark M. Churchland[3,4,5], Jennifer N. Gelinas[2,7*], Dion Khodagholy[1,8*]

1. Department of Electrical Engineering, Columbia University, New York, USA
2. Department of Biomedical Engineering, Columbia University, New York, USA
3. Department of Neuroscience, Columbia University, New York, NY, USA
4. Zuckerman Mind Brain Behavior Institute, Columbia University, New York, USA
5. Kavli Institute for Brain Science, Columbia University, New York, USA
6. Grossman Center for the Statistics of Mind, Columbia University, New York, USA
7. Department of Neurology, Columbia University Irving Medical Center, New York, USA
8. Department of Electrical Engineering, University of California, Irvine, California, USA

E-mail:
jng2146@cumc.columbia.edu
dk2955@columbia.edu
* Corresponding authors

Keywords: Conducting polymers, organic bioelectronics, anisotropic conductors, EMG

Abstract
Epidermal electrophysiology is a non-invasive method used in research and clinical practices to study electrical activity of the brain, heart, nerves, and muscles. However, electrode/tissue interlayer materials such as ionically conducting pastes can negatively affect recordings by introducing lateral electrode-to-electrode ionic crosstalk and reducing spatial resolution. To overcome this issue, we developed biocompatible, anisotropic-conducting interlayer composites (ACI) that establish an electrically anisotropic interface with the skin, enabling the application of dense cutaneous sensor arrays. We also microfabricated high-density, conformable electrodes that adhere to the ACI and follow the curvilinear surface of the skin. Our results show that ACI significantly enhances the spatial resolution of epidermal electromyography (EMG) recording compared to conductive paste, permitting the acquisition of single muscle action potentials with distinct spatial profiles. We validated our high-density EMG in developing mice, non-human primates, and humans. Overall, high spatial-resolution epidermal electrophysiology enabled by ACI has the potential to advance clinical diagnostics of motor system disorders and enhance data quality for human-computer interface applications.

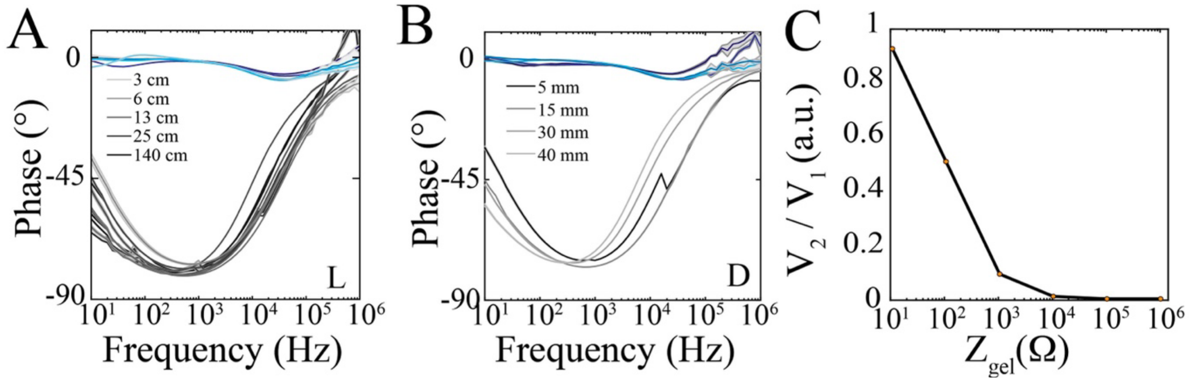

Supplementary Figure 1: Cutaneous tissue impedance spectroscopy.

A) Corresponding phase spectrum of the impedance spectrum shown in Figure 1B. Shaded areas represent standard errors (n = 3).

B) Corresponding phase spectrum of the impedance spectrum shown in Figure 1D. Shaded areas represent standard errors (n = 3).

C) Ratio of V_2 / V_1 as an indication of crosstalk as a function of interlayer bulk material impedance.

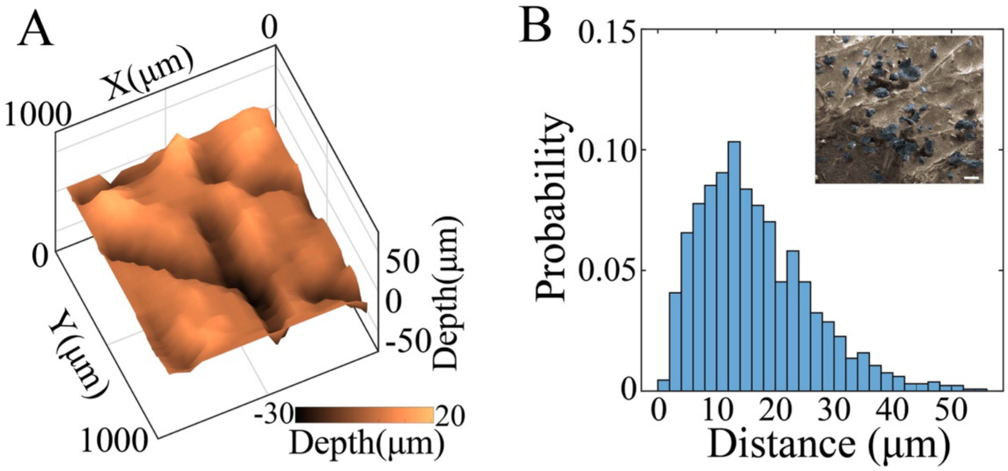

Supplementary Figure 2: Human skin surface roughness characterization.

A) Reconstructed topography of human skin based on 2D mechanical profilometry.

B) Histogram of PEDOT:PSS particulate distance measured by SEM (n = 52). Inset: tilted SEM of ACI particles applied on the surface of artificial skin. Scale bar, 50 μm.

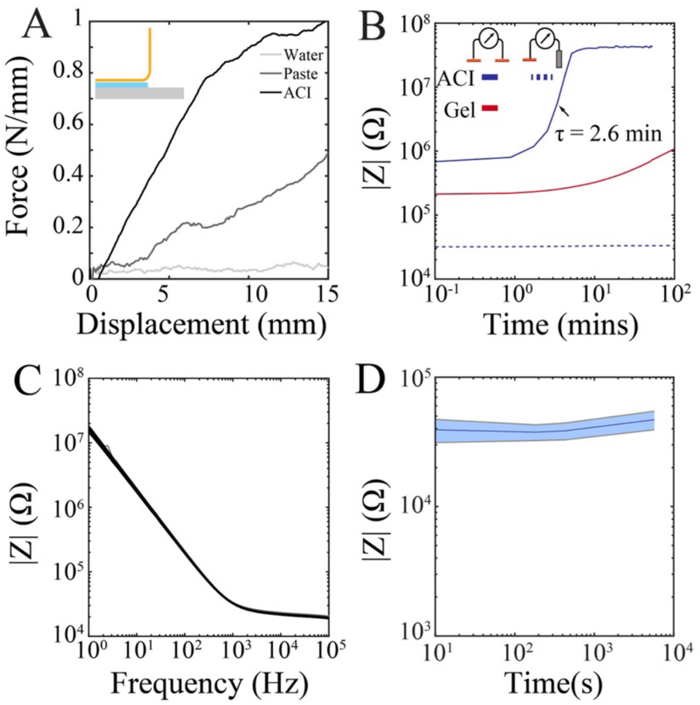

Supplementary Figure 3: ACI provides robust adhesion compared to commercial gels

A) Comparison of interfacial adhesion forces of electrode array with various interlayers.

B) Inter-electrode impedance of ACI (blue), conductive gel (red) and contact impedance with tissue with ACI (dashed blue) as a function of time with 400 × 400 μm^2 PEDOT:PSS electrodes, at 1KHz.

C) Time-elapsed EIS between electrode and tissue with ACI. The lighter shades indicate a longer elapsed time.

D) Time-elapsed tissue contact impedance of a NeuroGrid array. Shaded area represents standard error (N = 120).

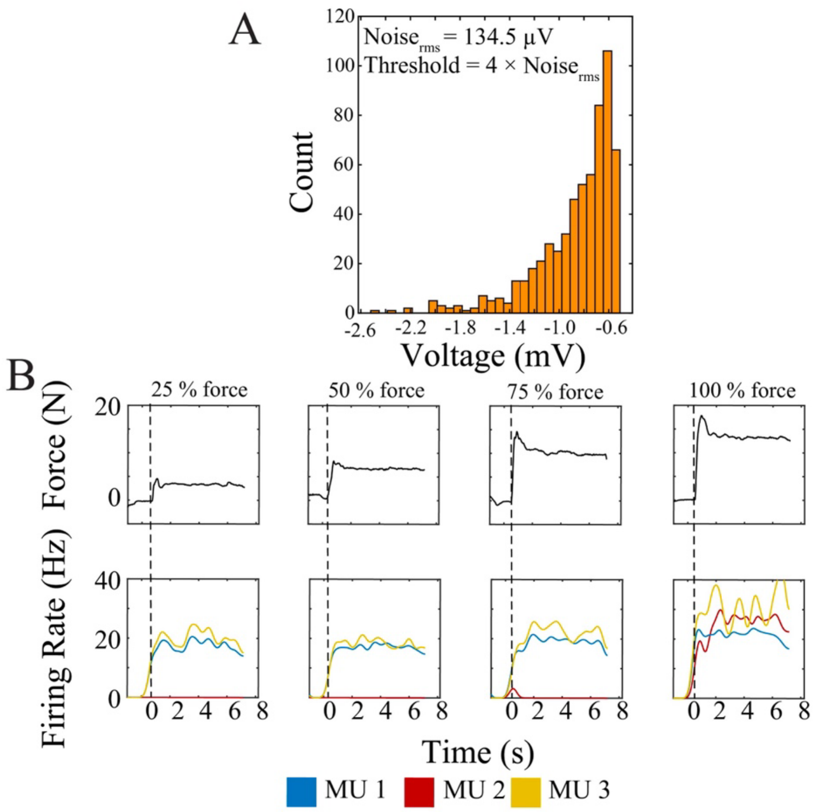

Supplementary Figure 4: Correlation of MUs and applied force during non-human primate experiments.

A) Histogram of amplitude of detected muscle action potentials during isometric task. The $Noise_{rms}$ is derived based on the median of absolute value of filtered traces.

B) MU firing rate temporally correlates with force level. Top panels: applied force measured by the load cell. Bottom panels: firing rate of decoded MUs as a function of behavioral onset elapsed time. Dashed line indicates the onset of force application.

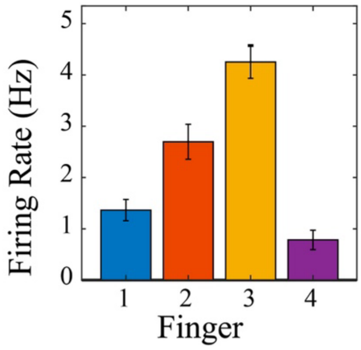

Supplementary Figure 5: Firing rate characterization of MUs as a function of finger movement.

Variability of single MU firing rate as a function of different finger presses. N = 912 spikes.
